# Editorial on the Research Topic of the Special Issue “Current Status of Acinetobacter Infections”

**DOI:** 10.3390/pathogens12020219

**Published:** 2023-01-31

**Authors:** Francesca Paola Nocera, Luisa De Martino

**Affiliations:** Department of Veterinary Medicine and Animal Production, University of Naples Federico II, Via F. Delpino 1, 80137 Naples, Italy

**Keywords:** *Acinetobacter* infections, *Acinetobacter baumannii*, antibiotic resistance, virulence factors, one health approach

Emerging bacterial infections will continue to be an important issue for public health, mostly because of the constant changes on our earth. The amount and speed of human mobility today, together with climate change and the loss of natural habitats, are probably the most evident manifestations of the current era of globalization. This has led to an increased risk of interaction between species, and within species. In this scenario, the *Acinetobacter* species, belonging to the *Acinetobacter calcoaceticus*-*Acinetobacter baumannii* (ACB) and the not-ACB complex, have become of particular concern in both veterinary and human medicine because of their antimicrobial resistance phenotypes.

*Acinetobacter* infections not only affect human health but have an impact on the health of domestic pet animals [1], with possible transmission of this microorganism between humans and pet animals [2], as well as of isolates from wildlife [3]. Therefore, a One Health approach is required, and this is valid for numerous infectious diseases, where the possibility of infectious agent movement between species, including human beings, is key. In particular, *Acinetobacter baumannii*, one of six bacterial pathogens of the ESKAPE group (the acronym ESKAPE includes six highly virulent and antibiotic-resistant nosocomial pathogens and precisely, they are *Enterococcus faecium*, *Staphylococcus aureus*, *Klebsiella pneumoniae*, *Acinetobacter baumannii*, *Pseudomonas aeruginosa*, and *Enterobacter* species), is primarily responsible for antibiotic-resistant infections and frequently associated with nosocomial infections, mainly in intensive care units, and has become a relevant threat for public health worldwide. Furthermore, recently its involvement in a case of coinfection with the SARS-CoV-2 virus has been reported [4,5]. Consequently, it is important to pay attention to bacterial co-infections in critical patients positive for SARS-CoV-2. On the other hand, bacterial co-pathogens are commonly identified in viral respiratory infections and are important causes of morbidity and mortality [6,7].

In the last twenty years, *A. baumannii*, highly virulent and representative of current outbreak strains, has become a major threat, not only as an important pathogen associated with nosocomial and community infections at various body sites (bloodstream, respiratory tract, urinary tract, surgical sites, and wounds) [8], but also because it poses a huge challenge in clinical settings due to its intrinsic and acquired antimicrobial resistance. One of the main research engines on *A. baumannii* diseases, especially for the problematic and worrying aspect regarding antimicrobial resistance, is of public health concern. Specifically, the knowledge of antimicrobial resistance gene acquisition, genomic mutations, or modifications in the expression of some genes plays a key role in the therapeutic success against multidrug-resistant (MDR) *A. baumannii* infections [9]. About this, the investigation of Cafiso et al. [10], in this Special Issue, on phenotypic and genomic characterization as well as on phylogenetic relationship in *A. baumannii* samples, showed different levels of colistin resistance (COL-R) and heteroresistance, suggesting more strategies that *A. baumannii* can use to become resistant. This leads to the strain definition as unstable or stable colistin-resistant and overall, to the so-called dynamism hypothesis of the COL-R stability acquisition.

In the field of studying genome dynamics, it is known that clustered, regularly interspaced, short palindromic repeat (CRISPR)/Cas systems, existing in most bacteria, may target either DNA or RNA to interfere with viruses, plasmids, prophages, or other chromosomally encoded sequences. Tyumentseva et al. [11], in their paper, demonstrated in *A. baumannii* the role of CRISPR/Cas systems in controlling antibiotic resistance gene transfer and acquisition and a clear association of MDR genotype/phenotype of *A. baumannii* with the type of its CRISPR/Cas system.

MDR clones circulated among humans, companion animals, and the environment, and in-depth genomic studies have been frequently carried out also in veterinary medicine. Different typing methods, including the conventional MLST typing, core-genome MLST (cgMLST), pan-genome analysis, and single nucleotide polymorphisms (SNP)-based molecular clock analysis have been employed for a comparative investigation on clinical canine, environmental and human reference strains [12]. This study demonstrated that animals and humans shared identical clones (ST2) and the same B-lactamase (blaOXA-66), underlining the importance of genomic investigation in the veterinary field in studying *A. baumannii*. It is clear that the surveillance of antimicrobial-resistant *A. baumannii* can increase awareness and help reduce the transmission of MDR *A. baumannii* infections in different surroundings, providing additional tools for genomic evolutionary studies.

Research on possible strategies to contain resistance is included in antimicrobial management programs and, of relevance are the statistically significant associations between different antimicrobials that can be derived from valuable study tools, such as predictive models [13].

Overall, this Special Issue collected papers on *A. baumannii* infections, focusing on new and useful diagnostic methods and highlighting the importance of studies with a One Health approach. Furthermore, it allowed us to underline the importance of genomic investigation combined with molecular insights with different techniques and statistical correlation studies in the field of antimicrobial resistance.

Taken together, these 11 contributions (seven original articles and four reviews) published in this Special Issue reinforce the importance of studies on this bacterium in various diseases of human and animal origin. 

Given the success of this Special Issue, a Pathogens webinar on 10 March 2022, online, entitled “Current Status of Acinetobacter Infections” was organized. It was a useful moment to set up an international scientific discussion. According to research, many people use webinars for information and updates on certain topics; hosting a webinar on this topic for a large audience (more than 80 people attended the webinar), that has allowed us to provide innovative knowledge that addresses the problems of antibiotic resistance of *Acinetobacter* infections, was a great pleasure for us.

We would like to thank the authors and reviewers of the papers in this Special Issue. We are also grateful to the Managing Editor, for her support, guidance, and help in coordinating the compilation of this Special Issue. Finally, this issue would not have achieved its high level of quality without the diligence and insights of all reviewers who provided high-quality reviews.
